# Application of coal-based solid waste artificial soil in the restoration of saline alkali land-taking the saline alkali land restoration of the Emao River in China as an example

**DOI:** 10.1016/j.heliyon.2024.e37095

**Published:** 2024-08-28

**Authors:** Haidong Zhao, Jiqing Chang, Zifeng Miao, Hongjie Kang, Jianbing Ji, Yu Luan, Zhen Lu, Yong Guo

**Affiliations:** a School of Chemistry and Chemical Engineering, Shanxi Datong University, Datong City, Shanxi Province, 037009, China; b Guangdong Changjiqing Fly Ash Research Institute, Heyuan City, Guangdong Province, 517000, China; c Shanxi Hongyu Solid Waste Comprehensive Utilization Research and Development Co., Ltd., Huairen City, Shanxi Province, 038300, China; d Shanxi Saixin Resource Recycling Technology Co., Ltd., Datong City, Shanxi Province, 037009, China

**Keywords:** Saline alkali land, Coal based solid waste, Artificial soil, Restoration

## Abstract

In this paper, coal-based solid waste, including fly ash, desulfurization gypsum, furnace bottom slag, and coal washing sludge in the ratio of 3:2:3:2 with a total of 200 tons/0.07 hm^2^, were used as the specialized main material for restoring the saline alkali land of Emao River in Huairen of China. The remediation effect and safety of solid waste artificial soil were evaluated by testing the soil samples before and after the remediation. The results showed that the pH value of the soil after remediation decreased from 9.98 to 7.60, which was close to the neutral value and suitable for crop growth. The total amount of water-soluble salts decreased from 8.30 g kg^−1^ to 4.80 g kg^−1^ with a decrease of 42.2 %. The organic matter increased from 6.5 g kg^−1^ to 39.1 g kg^−1^ with a 5-fold increase. Compared to the original soil, the heavy metal content in the restored soil did not increased, but instead decreased, indicating that the restoration technology was feasible and meets environmental requirements. Corn planting experiment results showed that corn's emergence rate in the original saline alkali soil was extremely low (about 1 %), while in the restored soil reached over 99 %. The average yield of corn in restoration field was 16.56 % higher than the average local yield level. The residual content of heavy metals and organic toxic substances in corn and potatoes grown on restored soil were analyzed, and the results showed that the detected heavy metal content was far lower than the standard values, and the residual organic toxic substances were basically not detected, indicating that the agricultural fruits grown on restored soil were safe, harmless, and edible. This approach could achieve large-scale consumption of coal-based solid waste, increase arable land, and reduce the cost of restoring saline alkali land.

## Highlights

• *Coal-based solid waste was used as the material for restoring saline alkali land.*
• *Heavy metals are not exceeded in the restoration of saline alkali land.*
• *The restoration demonstration field had reached excellent soil planting capacity.*
• *The agricultural fruits grown on restored soil are safe, harmless, and edible.*
• *This approach can achieve low-cost and large-scale restoration of saline alkali land.*

## 1 Introduction

Saline alkali land refers to soil where the accumulated salt content exceeds the level of normal cultivated soil and affects the normal growth of crops [1]. According to incomplete statistics, saline alkali land in the world accounts for approximately 25 % of the total land area [2]. In recent years, with the rapid development of agricultural production and the excessive use of fertilizers and pesticides, the world has added (1-15) × 10^6^ hm^2^ of saline alkali land every year [3,4]. China has a large number of contiguous saline alkali cultivated land resources, which are concentrated in four regions in northeast China (Jilin, Heilongjiang and Inner Mongolia), Qingxin (Qinghai, Xinjiang), northwest inland (Inner Mongolia, Ningxia, Gansu, etc.), and coastal and North China Plain (Hebei, Shandong, Jiangsu, etc.) [5]. The salinization of land leads to the inability to carry out normal agricultural production and reduces land productivity. Currently, about 20 % of the total area of lightly saline alkali land has been cultivated in China, and a large amount of medium to severe saline alkali land cannot be utilized, greatly wasting land resources [6,7]. Therefore, if the unsuitable medium and severe saline alkali land can be restored and meet crop growth requirements, it will not only significantly increase the cultivated land area but also ensure the demand for economic construction land through land replacement and transfer.

There are many restoration methods for saline alkali land, among which the use of solid waste such as fly ash, desulfurization gypsum, coal gangue, etc., for saline alkali land renovation has been reported [[8], [9], [10]]. This restoration method has low cost, fast effectiveness, significant improvement effect, and good application prospects. Mahale et al. reported that fly ash contains essential nutrients for plant growth, which could enhance the seed germination rate. Heavy metal test results showed that Cd, Cu, Fe, Mn, Mg Ni, Pb, and Zn were accumulated in the plants but at very low concentrations and below the permissible limits for human consumption [8]. Xu et al. applied desulfurization (fly ash and desulphurized gypsum) at an amount of 1 % of the total soil content, which had an effective effect in promoting the root growth of the crop and resulting in high yields [9]. Liu et al. by using FGD gypsum, could significantly reduce the pH and salt content in the 0–10 cm soil layer, and there was no significant change in the heavy metal content of the soil, indicating that FGD gypsum is safe for the improvement of saline soils [10].

China has the world's highest solid waste emissions (reaching 65 billion tons), and the storage and occupation of solid waste have brought tremendous pressure, causing serious environmental pollution. The utilization rate of solid waste in southern China is relatively high, while in northern China, it is relatively low (with a large solid waste base and a large amount of newly generated solid waste in the Tongshuo area in Shanxi province of China). There is an urgent need to find more solid waste market application areas to solve the current and future crises, and resource utilization and batch utilization are the main directions of attack. Therefore, it is imperative to develop a new application approach for solid waste, enhancing technological content, improving utilization efficiency, increasing economic added value, reducing environmental pollution, and comprehensively utilizing solid waste.

In this paper, the saline alkali land of the Emao River in Huairen of China was taken as the research object. Coal based solid waste, including fly ash, desulfurization gypsum, furnace bottom slag, and coal washing sludge, were used as the main material for soil restoration. The safety and remediation effect of solid waste-based artificial soil were evaluated by testing soil samples, crop emergence rate, growth status, and fruits before and after restoration, and comparing China's national soil standards and crop fruit detection standards. This approach can achieve batch consumption of bulk solid waste, with low cost and significant effect in restoring saline alkali land, and it can be widely promoted as a saline alkali land restoration method.

## 2 Materials and methods

### 2.1 Materials

Fly ash (Datong Tashan Power Plant, pH: 8.50, organic matter content: 19.8 g kg^−1^), desulfurization gypsum (Datong Tashan Power Plant, pH: 7.60 organic matter content: 15.7 g kg^−1^), furnace bottom slag (Huairen No.11 Middle School boiler, pH: 7.50 organic matter content: 4.2 g kg^−1^), coal washing sludge (Huairen Liudongying Village coal washing plant, pH: 6.80 organic matter content: 395 g kg^−1^), organic fertilizer (cow and sheep manure, pH: 6.40 organic matter content: 314 g kg^−1^), organic-inorganic soil modifier (self-made A, B, C reagent), which A mainly contains dihydrogen phosphate and heavy metal complexing agents, B is humic acid, C is inorganic binders.

### 2.2 Restoration methods for saline alkali land

In the experiment, the saline alkali land of Emao River was selected as the research object. The experimental field is located in the east of Liudongying Village, Maozao Town, Huairen City, Shanxi Province in China, which is a severely saline alkali wasteland with a salt content of 8.30 g kg^−1^ and a pH value of 9.98. The average annual temperature in Huairen City is 7.9 °C, the average temperature of the coldest month is −9.2 °C, and the average temperature of the hottest month is 22.7 °C; the average annual rainfall is 367.0 mm, of which the precipitation from April to September is 323.9 mm; the average annual relative humidity is 48 %, and the number of sunshine hours for the whole year is 2862.0 h. According to statistics, Liudongying Village currently has over 53 hm^2^ of severely saline alkali land of the same kind. Pine trees are mainly planted, but it is difficult to survive. The mortality rate in the year of planting reaches 30–40 %, and in the later stage, it basically withered and died. The planting area selected for this experiment was about 0.07 hm^2^, and the experimental field area for comparison was about 0.03 hm^2^.

Coal-based solid waste such as fly ash, desulfurization gypsum, furnace bottom slag, and coal washing sludge were used as the main materials for soil restoration. Key organic-inorganic soil modifiers (self-made ABC reagent) were added, where A was the activator (activating trace elements in solid waste, allowing plants to absorb them, increasing nutrients, and passivation of heavy metal ions), and B was the regulator (further regulating the pH value and nutritional composition of the mixed artificial soil), C was an intermediate (to solve the problem of soil stacking density and ensure good connection between the restored soil and the original soil), thus obtaining solid waste artificial soil. Then, the artificial soil was thoroughly mixed with the original saline alkali soil to complete the restoration.

Solid waste-based artificial soil was prepared by mixing coal-based solid waste with fly ash (60 tons), desulfurization gypsum (40 tons), furnace bottom slag (40 tons), coal washing sludge (60 tons), and cow and sheep manure (5 tons) together. Firstly, a total of 205 tons of artificial soil was spread flat on the saline alkali land. A total of 1.5 kg of organic-inorganic soil modifiers were diluted in 500 kg of water and sprayed onto the surface of the artificial soil. The soil was then plowed longitudinally for over 30 cm using a cultivator. Then, the same amount of organic-inorganic soil modifier was resprayed, with a horizontal depth of more than 30 cm, to ensure a uniform mixture of artificial and original soil. After using a rotary tiller to level the land, crops can be planted. After restoration, the soil salinity decreased to 4.8 g kg^−1^, the pH value was 7.60, and all heavy metals met the standard requirements for planting soil. Then, various sparse vegetables and crops can be planted. This field experiment started in June 2022 and is currently in its third year of planting. The crop is growing well and there have been no problems with the soil returning to salinity.

### 2.3 Analyzing methods for soil and crop samples

After the soil was restored, we mainly planted corn and potatoes. By checking soil samples, crop emergence rate, growth status, etc., before and after restoration to evaluate the remediation effect and the safety of solid waste-based artificial soil. The sampling methods for soil and plants followed standardized procedures to ensure data accuracy and reproducibility. For soil samples, a sampling depth of 0–20 cm was selected as the surface soil layer, as this layer typically contains the richest biological activity and most of the nutrients required for plant growth. Five sampling points were established, and after thorough mixing, the soil samples were collected and sent for testing. In studies requiring analysis of the relationship between soil depth and pH value, soil samples were also collected from depths of 20–40 cm, 40–60 cm, or below 60 cm. For sampling of corn and potatoes, a random sampling method was employed, and the collected samples were immediately placed in sealed plastic bags and sent to the laboratory for further analysis and processing.

The samples of soil, corn and potatoes were analyzed by the Shanxi Provincial Quality Inspection Bureau (the organization has MA testing qualification certification) in accordance with relevant Chinese national standards (GB 15618-2018 Soil Risk Control Management Standard, GB2762-2022 Food Pollutant Limits). The soil detection items including available phosphorus (NY/T 1121.7–2014), total water-soluble salt (NY/T 1121.16–2006), total nitrogen (NY/T 1121.24–2012), organic matter (NY/T 85–1988), available potassium (NY/T889-2004), pH value (NY/T 1377–2007), and heavy metal content of total mercury (GB/T 22105.1-2008), total arsenic (GB/T 22105.2-2008), cadmium (GB/T 17141-1997), plumbum (GB/T 17141-1997), zinc (HJ 491–2019), and chromium (HJ 491–2019). The heavy metal content in corn and potatoes were analyzed using the Chinese national standard method, and the detection items were total mercury (GB 5009.17-2021), total arsenic (GB 5009.11-2014), cadmium (GB 5009.15-2014), plumbum (GB 5009.12-2017), zinc (GB 5009.14-2014), and chromium (GB 5009.123-2014). All experimental data were averaged after accurate testing by taking three samples.

## 3 Results and discussion

### 3.1 Formulation design of salt alkali soil restoration

The design of the formula for restoring saline alkali soil adopted the small-scale method in a laboratory. Various raw materials (raw saline alkali soil, fly ash, desulfurization gypsum, furnace bottom slag, coal washing sludge, organic fertilizer, and organic-inorganic soil modifier) were mixed uniformly in a certain proportion. The pH value, total water-soluble salt content, and organic matter were tested, and the optimal ratio was determined based on the measured data. The results were shown in Table 1.

**Table 1:** Formulation Design of salt alkali soil restoration.

	original saline alkali soil	fly ash	desulfurization gypsum	furnace bottom slag	coal washing sludge	organic fertilizer (tons/0.07 hm^2^)	soil modifier (kg/0.07 hm^2^)	pH value	Total water soluble salt g·kg^−1^	Organic matter g·kg^−1^
1	40 %	18 %	12 %	12 %	18 %	5	3	6.81 ± 0.05	3.92 ± 0.15	43.0 ± 0.10
2	50 %	15 %	10 %	10 %	15 %	5	3	7.13 ± 0.05	4.34 ± 0.15	41.2 ± 0.10
3	60 %	12 %	8 %	8 %	12 %	5	3	7.42 ± 0.05	4.51 ± 0.15	40.8 ± 0.10
4	70 %	9 %	6 %	6 %	9 %	5	3	8.05 ± 0.05	5.14 ± 0.15	38.3 ± 0.10
5	60 %	12 %	8 %	8 %	12 %	5	2	8.15 ± 0.05	4.68 ± 0.15	40.2 ± 0.10
6	60 %	12 %	8 %	8 %	12 %	5	4	6.53 ± 0.05	4.30 ± 0.15	41.5 ± 0.10
7	60 %	12 %	8 %	8 %	12 %	3	3	7.71 ± 0.05	4.55 ± 0.15	31.9 ± 0.10
8	60 %	12 %	8 %	8 %	12 %	7	3	7.25 ± 0.05	4.39 ± 0.15	48.6 ± 0.10

In experiments 1–4, fly ash, desulfurization gypsum, furnace bottom slag, and coal washing sludge were used as the main materials for restoration soil, with a relative ratio of 3:2:2:3. The amount of fixed organic fertilizer used was 5 tons/0.07 hm^2^, and the amount of soil modifier used was 3 kg/0.07 hm^2^. The optimal soil ratio was determined by changing the amount of original saline alkali soil used. The results showed that as the amount of original saline alkali soil used increased from 40 % to 70 %, the pH value of the mixed soil sample gradually increased. The content of water-soluble salts gradually increased, mainly due to the decrease in the amount of solid waste added, which weakens the dilution effect. The change in organic matter content were gradually decreased with the proportion of coal slurry usage decreasing, mainly due to the large amount of organic matter such as humic acid in coal slurry. From the changes in pH value, water-soluble salt content, and organic matter content indicators, it could be seen that when the original saline alkali soil was used at 60 %, the pH value was close to neutral, and the water-soluble salt content and organic matter content also reached a relatively appropriate level. Experiments 3, 5, and 6 investigated the effect of soil modifier dosage on soil remediation. The results showed that when the original saline alkali soil dosage was 60 %, the pH value changed from high to low with an increase in soil modifier dosage, and the effect was significant. At the same time, soil modifiers also had a good effect on reducing water-soluble salts, but their contribution to organic matter was relatively small. In the experiment, selecting a 3 kg/0.07 hm^2^ soil modifier dosage could achieve good indicators. Experiments 3, 7, and 8 investigated the effect of organic fertilizer dosage on soil remediation. The results showed that organic fertilizer could effectively increase organic matter content and regulate soil pH, mainly due to the presence of a large amount of humic acid in organic fertilizer. Considering the cost and remediation effect, a dosage of 5 tons/0.07 hm^2^ of organic fertilizer was selected, and the remediation index was more suitable. In summary, this experiment selected a ratio of 60 %: 12 %: 8 %: 8 %: 12 % for the original saline alkali soil, fly ash, desulfurization gypsum, furnace bottom slag, and coal washing sludge. The fixed organic fertilizer usage was 5 tons/0.07 hm^2^, and the soil modifier usage was 3 kg/0.07 hm^2^. The restored soil pH value was 7.42 ± 0.05, the water-soluble salt content was 4.51 ± 0.15 g kg^−1^, and the organic matter content was 40.8 ± 0.10 g kg^−1^, which met the requirements of planting soil.

### 3.2 Comparison between original saline alkali soil and restored soil

**Fig. 1:**
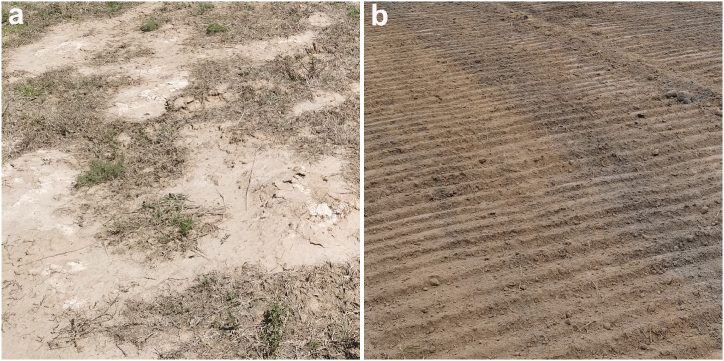
Comparison images of saline alkali soil layers before (a) and after (b) restoration.

The formula for soil restoration in saline alkali land was determined based on the experimental results of the soil testing formula. The comparative test results of surface soil samples before and after restoration showed that the total amount of water-soluble salts in the soil before restoration was 8.30 g kg^−1^, with a pH value of 9.98, a typical heavy saline alkali land. After using solid waste artificial soil for restoration, the total amount of water-soluble salts in the soil decreased to 4.80 g kg^−1^, and the pH value decreased to 7.60, approaching neutrality. Fig. 1 showed the comparison before and after the restoration of saline alkali land. From Fig. 1a, it could be seen that the soil of the original saline alkali land was hard and compacted. Some areas were covered with a layer of white saline alkali substance on the surface, which basically could not grow plants except that some salt alkali tolerant weeds. After artificial soil restoration, the surface soil approached the normal color of cultivated land, and the soil layer was loose suitable for cultivation (Fig. 1b).

**Fig. 2:**
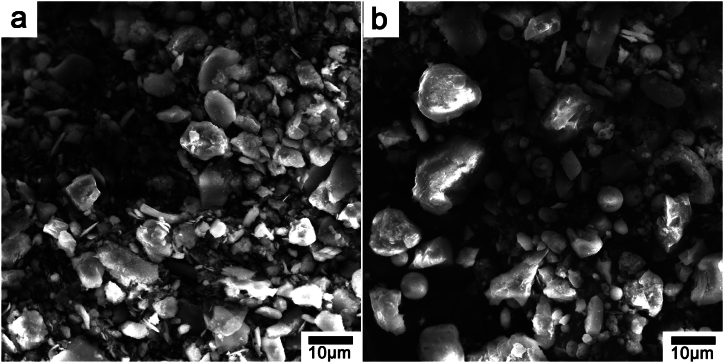
SEM images of saline alkali soil before (a) and after (b) restoration.

**Fig. 3:**
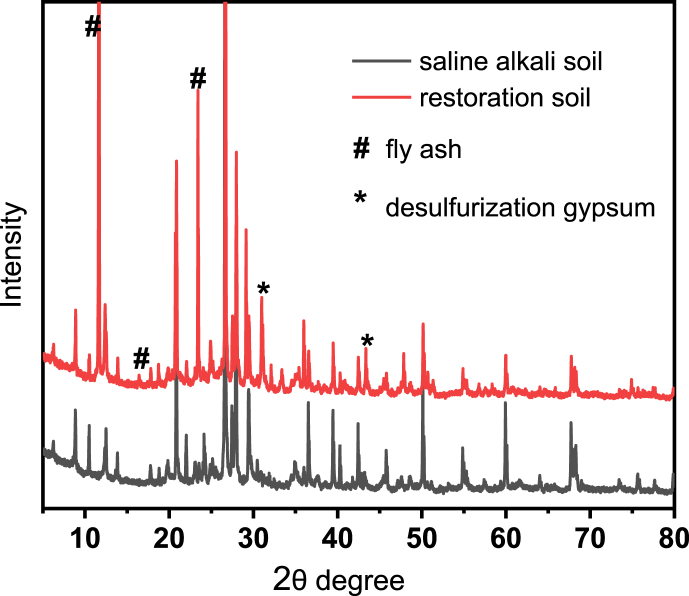
The XRD spectra before (black line) and after (red line) restoration of saline alkali soil.

Microscopic morphology analysis was conducted on the soil samples before and after restoration, and the SEM results were shown in Fig. 2. From Fig. 2a, it was evident that the microstructure of the original saline alkali soil exhibited a disordered structure and irregular distribution. After adding solid waste, the added fly ash glass bead-like structure (diameter 3–5 μm) and desulfurization gypsum columnar structure (size about 10 μm) can be clearly seen in the restored soil. The XRD spectrum (Fig. 3) further confirmed the addition of materials such as fly ash and desulfurization gypsum to the restored soil. The peaks with diffraction angles of 11.570, 16.540, 23.510, 31.010, and 43.460 could be attributed to the diffraction peaks of fly ash and desulfurization gypsum. At the same time, the XRD diffraction peaks were sharp, indicating that the various components of artificial soil were relatively stable and had good crystal structure.

Currently, China's soil environmental quality standard for soil pollution risk control of agricultural land (trial) (GB 15618-2018) adopts a dual line management approach of risk screening values and risk control values based on the total amount of heavy metals in soil [11,12]. The national standard methods were used to analyze the composition of soil samples before and after restoration. The detection items were available phosphorus, total water-soluble salt, total nitrogen, organic matter, available potassium, pH value, and heavy metal content (total mercury, total arsenic, cadmium, plumbum, zinc, and chromium). The results were shown in Table 2. Comparing the detection values of soil samples before and after restoration, it could be seen that the restored soil had greatly improved in all indicators. The pH value of the acidity and alkalinity decreased from 9.98 to 7.60, approaching a neutral value and suitable for crop growth. The total amount of water-soluble salts decreased from 8.30 g kg^−1^ in severely saline soil to 4.80 g kg^−1^ with a decrease of 42.2 %. The organic matter increased from 6.5 g kg^−1^ to 39.1 g kg^−1^ with a 5-fold increase. And the nitrogen, phosphorus, and potassium in the restored soil had been effectively improved.

**Table 2:** Results of before and after the restoration of saline alkali soil testing by using the national standard method.

	Items	Unit	Values of before remediation	Values of after remediation	National standard values
1	Available phosphorus	mg·kg^−1^	3.4	17.4	/
2	Total water soluble salt	g·kg^−1^	8.30	4.80	Non saline (<1g·kg^−1^); Mild saline (1–3g·kg^−1^); Moderate saline (3–6g·kg^−1^); Severe saline (6–10 g kg^−1^); Saline soil (>10 g kg^−1^)
3	Total nitrogen	g·kg^−1^	0.57	0.66	/
4	Organic matter	g·kg^−1^	6.5	39.1	/
5	Available potassium	mg·kg^−1^	98	117	/
6	pH value	/	9.98	7.60	＜6.5 acidity 6.5–7.5 neutral >7.5 alkalinity
7	Total Mercury	mg·kg^−1^	0.13	0.12	2.4
8	Total Arsenic	mg·kg^−1^	8.45	7.44	30
9	Cadmium	mg·kg^−1^	0.079	0.066	0.3
10	Plumbum	mg·kg^−1^	12.6	2.57	120
11	Zinc	mg·kg^−1^	59	51	250
12	Chromium	mg·kg^−1^	55.0	50.8	200

For the large-scale field experiment (for 0.07 hm^2^ or 667 m^2^), the solid waste-based artificial soil was prepared by mixing coal-based solid waste with fly ash (60 tons), desulfurization gypsum (40 tons), furnace bottom slag (40 tons), coal washing sludge (60 tons), and cow manure (5 tons) together. Then, a total of 205 tons of artificial soil was spread flat on the saline alkali land. Organic-inorganic soil modifiers A (activator, pH:1.70), B (regulator, pH:3.80), C (intermediate, pH: 7.90) were sprayed onto the surface of the artificial soil. The soil was then plowed for more than 30 cm using a cultivator to ensure an even mix. The pH value was adjusted with coal washing sludge (pH: 6.80), organic fertilizer (pH: 6.40), organic-inorganic soil modifier (self-made A pH:1.7 and B pH:3.8); after mixed together, the pH value was decreased from 9.98 to 7.60. The various types of solid waste used contain large amounts of residual carbon and inorganic minerals that can be used as organic fertilizer. In particular, coal washing sludge and cow manure generally contain between 300 and 400 g kg^−1^ of organic matter; it is reasonable to increase the organic matter content. For the water-soluble salt content was decreased from 8.30 g kg^−1^ to 4.80 g kg^−1^, attributed to the low salt content of each of the solid waste feedstocks used, bulk blending provided effective dilution, thus reducing the salt content of the soil. In addition, Ca^2+^ in desulphurized gypsum can effectively reduce free Na^+^ in saline soils, thus further reducing the soluble salt content and making the salt content in the remediated soil suitable for crop growth [10].

Compared to the original soil, the heavy metal content in the restored soil did not increased but instead decreased. The main reasons for the decreased in heavy metal content in the restored soil were: 1) the low content of heavy metals in the used solid waste, and the total content decreased after mixing with the original saline alkali soil; 2) Heavy metals in solid waste were in a passive and encapsulated state. For example, fly ash is formed through high-temperature combustion, during which all metals are contained in glass microspheres and difficult to separate out; 3) In addition, soil activator A material could passivate heavy metals and inhibit their precipitation. By comparing the results of national standard testing before and after soil restoration in Table 2 with the corresponding national standard values, it was found that all indicators of the restored soil meet the requirements of the relevant Chinese standard GB 15618-2018 soil risk management standard. This indicated that using coal-based solid waste for soil restoration technology was feasible and meets environmental protection requirements.

**Fig. 4:**
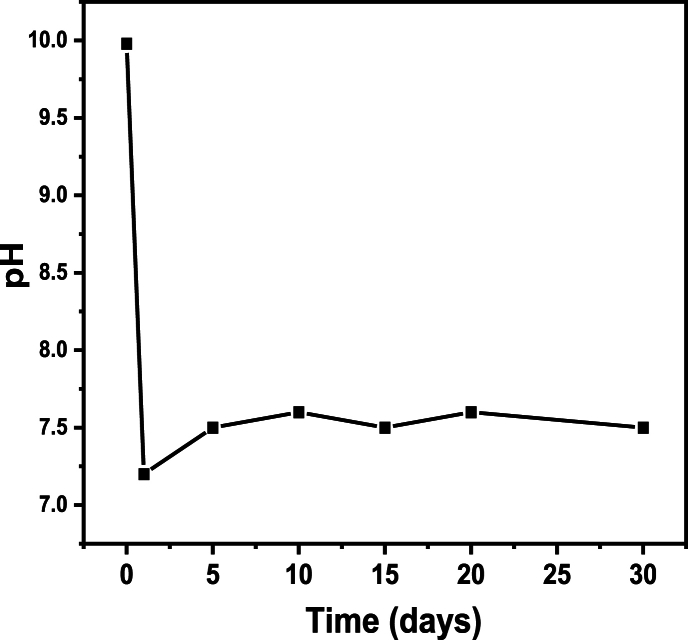
Relationship curve between pH values and time for soil restoration.

The organic-inorganic soil modifiers used in the experiment were composed of soil activator (A), soil regulator (B), and intermediate (C) mixed in a certain proportion. Soil activators mainly use weakly acidic or weakly alkaline substances to activate trace elements in solid waste, allowing plants to absorb and increase nutrients while also playing a role in passivating heavy metal ions. Regulators mainly used phosphates (such as calcium hydrogen phosphate, calcium dihydrogen phosphate, diammonium hydrogen phosphate, calcium magnesium phosphate fertilizer, and phosphorus containing sludge) and humic acid substances to further regulate the pH value and nutritional composition of mixed artificial soil, creating an environment suitable for crop growth. The intermediate uses inorganic binders to solve the problem of soil stacking density, ensuring a good connection between the repaired soil and the original soil, and improving the soil's water retention capacity. In addition, soil intermediates acted as salt inhibitors by disrupting the structure of compacted soil and capillary phenomena, blocking the regeneration of salt and alkali in the cultivated layer. Its pH regulation ability and salt alkali resistance effect were outstanding through experimental verification. After being sprayed with a modifier and mixed evenly, the pH value of the artificial soil with solid waste decreased from 9.98 to 7.60, reaching neutrality and suitable for crop growth. Through observation of two growth cycles, no alkali or salt layer backflow phenomenon was observed, indicating that soil modifiers have the effect of causing excessive salt alkali deposition and blocking the regeneration of salt alkali in the cultivated layer. Fig. 4 showed the variation curve of pH values of surface soil over time. After the soil was restored, the pH value first dropped sharply from 9.90 to 7.20 and then remained basically unchanged at 7.50–7.60, further confirming the blockage of the saline alkali layer and the absence of saline alkali return after soil restoration.

**Fig. 5:**
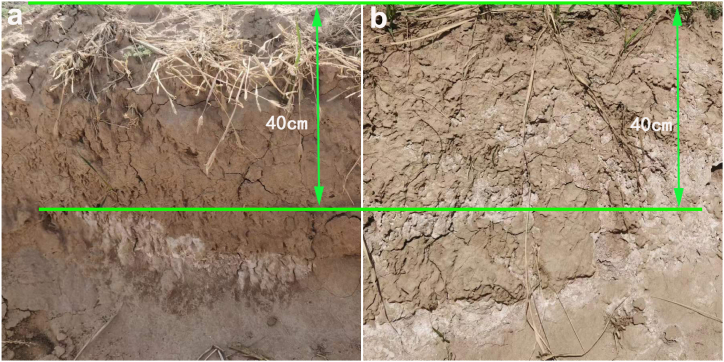
Distribution of saline in soil layer: after (a) and before (b) restoration of saline alkali soil.

By observing the isolation ditch excavated on the side of the experimental field, it could be clearly seen that the salt layer was blocked, as shown in the distribution of soil layers in Fig. 5. The saline alkali soil layer was compressed and isolated below 40 cm (Fig. 5a). The pH of the soil surface above 40 cm was about 7.60; the pH suddenly changed from 40 cm to 60 cm to above 9.9, and the pH reached above 11.0 below 60 cm. The saline alkali layer was located below the root system of crop growth and had a relatively small impact on crop growth. By comparison, the white saline alkali layer of the original saline alkali soil in Fig. 5b was distributed throughout the entire crop growth layer, with a pH above 9.50, indicating that the restored soil layer's salt isolation effect was absolutely excellent.

The organic matter content in soil is generally positively correlated with soil quality [13], and soil salt content is one of the important indicators to determine whether the soil is saline alkali. Due to the chelation, adsorption, and ion exchange effects of humic acid on salt alkali ions, it can promote the formation of large aggregate structures in soil particles, reduce the capillary phenomenon of fine soil particles, and reduce the salt carried to the soil surface by water evaporation [14]. Soil mineral particles are the basis for the formation of aggregates, and they have strong adsorption and binding effects with organic matter [[15], [16], [17]]. Due to the negative charge of humic acid colloids, using humic acid to restore soil can increase the soil's adsorption of cations, play a role in salt absorption, and block salt content. According to the report, humic acid can significantly reduce the total salt content in saline alkali soil, reducing it by 29.86 % compared to the original soil [18]. The use of coal washing sludge in solid waste in this experiment played the role of humic acid. Coal washing sludge contains a large amount of organic matter, which will be decomposed by microorganisms after being left for a period of time. The decomposed substance is humus, which will be hydrolyzed under certain conditions and release humic acid. In the experiment, adding coal washing sludge to the restored soil increased the organic matter by about 5 times, which not only adding nutrients to the soil but also reducing salinization.

Furthermore, adding a large amount of solid waste artificial soil to saline-alkali soil may have an impact on soil microbial metabolism. As pH value, soluble salt content, and nutrient content change, the soil microbial community will also respond accordingly. Therefore, when implementing the soil improvement measures, it is necessary to fully consider their potential impact on soil microbial metabolism and take reasonable measures to mitigate adverse effects, thereby promoting the healthy development of the soil ecosystem.

### 3.3 Comparison of crop growth between original saline alkali soil and restored soil

**Fig. 6:**
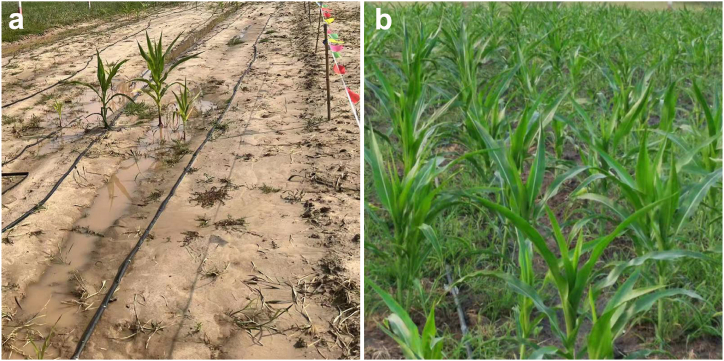
The emergence and growth condition of corn after 30 days of planting: planted in before (a) and after (b) restoration of saline alkali soil.

In the experiment, 0.03 hm^2^ of the original saline alkali soil was selected as a comparison field, and 0.07 hm^2^ of the restored soil was planted. Mechanized corn planting was used, and drip irrigation was also laid for later irrigation. Fig. 6 showed the emergence and growth of corn after 30 days of planting. As shown in Fig. 6a, the germination rate of corn in saline alkali soil was extremely low (about 1 %). Due to soil compaction, it was difficult for corn seeds to break through the soil and emerge after germination. The accumulated water on the ground was the same day's rainfall, and the soil was too compacted and dense, making it difficult for rainwater to penetrate. The growth of corn seedlings was malnourished, and yellow leaves appeared. In contrast, the corn planted in the restored soil had a seedling emergence rate of over 99 %, vigorous growth, and higher plant height than in the original saline alkali soil (Fig. 6b). The soil quality of the restored soil was loose, with strong permeability and water retention. In the experiment, there was continuous heavy rain, and there was no water accumulation on the surface. Rainwater could quickly penetrate into the soil layer. The water stored in the restored soil during drought periods could continuously supply water to crops and had excellent water retention and drought resistance. This can be attributed to the porous structure of the solid waste in the restored soil, which can effectively store and regulate water and nutrients, ensuring the growth of crops [[19], [20], [21], [22]].

After two years of planting, the yield of corn planted in the demonstration field was tested in October 2023. According to expert sampling and production measurement calculations, the average yield of corn per hm^2^ in the demonstration field of saline alkali land restoration was 12,238.5 kg, which was 16.56 % higher than the average local yield level of 10,500 kg per hm^2^. And compared to untreated saline alkali land, there was almost no harvest of corn. This indicated that the restoration demonstration field had achieved excellent soil planting capacity.

### 3.4 Testing results of crops planted in restored soil

The heavy metal content in corn and potatoes planted in the restored soil were analyzed using the Chinese national standard method. The detection items were total mercury, total arsenic, cadmium, plumbum, zinc, and chromium, and the results were shown in Table 3. It could be seen that the heavy metal content detected in crops were far lower than the national standard requirements, and some have not even been detected. The residual organic toxic substances in corn and potatoes grown in the restored soil were tested. The testing items for corn included deoxynivalenol, zearalenone, aflatoxin B1, omethoate, phorate, glyphosate, methomyl, carbendazim, methyl isocarbophos, and isocarbophos. The potato testing items included dichlorvos, feniconazole, avermectin, parathion, methyl parathion, phorate, and methamidophos. All of the residual organic toxic substances were not detected, indicating that the crops grown in the restored soil were safe, harmless, and edible.

**Table 3:** The testing results of corn and potatoes planted in restored soil by using the national standard method.

	Testing Items		Result of corn	Result of potatoe	Standard values
1	Mercury	mg·kg^−1^	0.0066	0.0036	0.02/0.01
2	Arsenic	mg·kg^−1^	0.034	0.019	0.7/0.2
3	Cadmium	mg·kg^−1^	negative	0.018	0.05/0.2
4	Plumbum	mg·kg^−1^	0.044	negative	0.4/0.4
5	Zinc	mg·kg^−1^	32.57	7.27	50/15
6	Chromium	mg·kg^−1^	0.28	0.12	1.0/0.5

I.

## 4 Conclusion

In this paper, the Huairen Emao River saline alkali land was successfully restored by artificial soil based on coal-based solid waste, leading to the following conclusions.

1) Coal-based solid waste, including fly ash, desulfurization gypsum, furnace bottom slag, and coal washing sludge, could be used as the main materials for restoration soil.
2) The pH value of the soil after remediation decreased from 9.98 to 7.60, which was close to the neutral value and suitable for crop growth. The total amount of water-soluble salts decreased from 8.3 g kg^−1^ in heavily saline soil to 4.8 g kg^−1^ with a decrease of 42.2 %. The organic matter increased from 0.65 % to 3.91 %, with a 5-fold increase.
3) After restoration, the saline alkali land had no excessive heavy metal ions, could be directly used for crop cultivation, and absolutely met the national standard requirements.
4) The emergence rate of restored soil crops was high and grew very well. The checking results of corn and potatoes showed that the crop fruits had no excessive heavy metals, no residual organic toxic substances, and meet the requirements of edible standards.
5) This approach can achieve batch consumption of coal-based solid waste, increase arable land, and reduce the cost of restoring saline alkali land. It can provide technical support for large-scale saline alkali land restoration promotion.
6) The limitation of using this method to restore saline-alkali land lies in its primary focus on areas with low groundwater levels, excluding coastal and wetland saline-alkali land. Furthermore, before restoration, all solid waste raw materials and artificial soil need to undergo professional testing to ensure that they do not cause pollution to the soil and groundwater. Therefore, the technical requirements are relatively high in the process of promoting this technology.

## Data availability statement

Data included in article.

## CRediT authorship contribution statement

**Haidong Zhao:** Writing – review & editing, Writing – original draft, Methodology. **Jiqing Chang:** Methodology, Investigation, Data curation. **Zifeng Miao:** Project administration, Investigation. **Hongjie Kang:** Investigation, Formal analysis. **Jianbing Ji:** Validation, Funding acquisition. **Yu Luan:** Validation, Funding acquisition. **Zhen Lu:** Writing – review & editing, Writing – original draft, Visualization, Investigation, Data curation. **Yong Guo:** Supervision, Project administration.

## Declaration of competing interest

The authors declare that they have no known competing financial interests or personal relationships that could have appeared to influence the work reported in this paper.
